# Hospitalizations and associated costs among older adults receiving regular home care services

**DOI:** 10.1007/s40520-026-03455-8

**Published:** 2026-07-11

**Authors:** Eeva Björkstedt, Ari Voutilainen, Kati Päivärinta, Virva Hyttinen-Huotari, Johanna Jyrkkä, Pekka Mäntyselkä, Eija Lönnroos

**Affiliations:** 1 Wellbeing Services County of South Savo, Porrassalmenkatu 35-37, Savonlinna, Mikkeli, FI-50100 Finland; 2 Institute of Public Health and Clinical Nutrition, Faculty of Health Sciences, University of Eastern Finland, Kuopio, Finland; 3 Department of Health and Social Management, University of Eastern Finland, Kuopio, Finland; 4 Research and Information Section, Finnish Medicines Agency FIMEA, Kuopio, Finland; 5 Teaching Clinic, Kuopio University Hospital, Wellbeing Services County of North Savo, Kuopio, Finland

**Keywords:** Hospitalization, Home care, Older adults, Health care costs, Functioning

## Abstract

**Background:**

Healthcare costs increase rapidly in aging societies. Research on service use is warranted in this context.

**Aims:**

To examine predicting factors and costs associated with hospitalizations among home care clients aged ≥ 65 years.

**Methods:**

Baseline data collection included demographic characteristics, diagnoses, morbidity (Charlson Comorbidity Index, CCI), basic and instrumental activities of daily living (BADL, IADL), Timed up and Go (TUG), Mini-Mental State Examination (MMSE) and health-related quality of life (EQ-5D-3 L). Univariate and multivariate logistic regressions were used to identify factors predicting hospitalization and gamma regression with a log link to analyze cumulation of inpatient days during one-year follow-up. Hospitalization costs were calculated using national unit costs of healthcare.

**Results:**

During follow-up, 176 (60%) of 293 regular home care clients were hospitalized. In univariate analyses, hospitalizations were statistically significantly associated with CCI, heart failure, peripheral vascular disease, renal failure, BADL, IADL and Eq. 5D3L. In multivariate analysis, only higher CCI (*p* = 0.008) predicted increased odds for hospitalization. Several diseases, number of medicines, BADL, IADL and MMSE were associated with inpatient days in univariate analyses. MMSE alone remained significant (*p* = 0.039) in multivariate analysis, higher scores predicted fewer days. Total hospitalization costs were €2,370,910, with €1,267,519 attributable to primary care and €1,103,391 to secondary care. Costs in the cohort were €9,225 per person-year.

**Discussion:**

Over half of regular home care clients required hospitalization with substantial costs during one year. Greater comorbidity burden predicted hospitalizations and cognitive impairment increased inpatient days.

**Conclusions:**

Optimal disease management and maintenance of functioning should be prioritized in home care.

## Introduction

Home care clients are a growing and vulnerable patient group with multimorbidity and complex needs for health and social services. Home care is considered a possible strategy to reduce the use of other health and social care services; [1–3] yet unplanned hospitalizations are nevertheless common among home care clients. According to an earlier Finnish study, 43% of new home care clients were hospitalized during a one-year follow-up [4, 5].

Higher total health care service use, comorbidity and cognitive impairment have been associated with higher hospitalization costs [6, 7]. Hospitalization costs peak during a patient’s last year of life but may also decrease with age and shift to long-term care [8]. Hospitalizations among older people are also associated with many negative outcomes such as rapid functional decline and death [9, 10].

Hospitalizations of older people are often due to acute exacerbations of chronic diseases, infections or injuries [1, 4, 11]. Earlier studies among home care clients or home-dwelling older people also indicate that many health-related and social factors, such as previous coronary artery disease, congestive heart failure, Parkinson’s disease, a history of falls, cognitive impairment, housing-related problems, health-related quality of life (HRQoL) and poor self-rated health, are risk factors for hospitalizations [1, 5, 11–21]. One major risk factor for hospitalizations among older people is functional disability, which also is the main prerequisite for receiving home care services [15, 18, 22, 23].

In Finland, home care and nursing services for older adults were previously arranged by municipalities. Since 2023, they have been provided by wellbeing services counties. Population structure of Finland is aging rapidly, and strategies to reduce the use of expensive care services, such as residential, nursing home or hospital care, are a particular of focus of health policy. In Finland, hospital care is organized at the primary, secondary, and tertiary care levels, and for example, rehabilitation of older adults is often provided in primary care hospitals. In the Finnish healthcare system, the majority of hospitalization costs are publicly funded through taxation, while patient fees account for approximately 4% of the total costs.

Previous studies on hospitalizations have considered newly enrolled home care clients or community-dwelling older adults without home care services. In this study, hospitalizations and number of inpatient days were examined among regular home care clients during one-year follow-up.

The primary diagnoses for hospitalizations and care settings were analyzed as well. Little is known about the costs of hospitalizations among home care clients; to fill the gap, we analyzed the costs of hospital care. Findings could support future home care planning and priority setting.

## Materials and methods

### Study design and participants

This study was a part of the Finnish Interprofessional Medication Assessment (FIMA) project. A detailed description of the FIMA study design has been reported previously [24–26]. The FIMA study evaluated a physician-led interprofessional medication assessment in comparison with usual care within public home care services across five regions in Finland. Eligibility criteria included age ≥ 65 years, receiving public home care services on regular basis, and at least one of the following: daily use of ≥ 6 medicines, dizziness, orthostatic hypotension or has experienced a fall during the preceding 12 months.

The present study was a subgroup analysis of the FIMA study. The participants were established home care clients residing in the Savonlinna region, located in Eastern Finland. The population aged 65 and over living in the Savonlinna region, East Savo Hospital District, was 12 400 at the end of the year 2015 [27]. Approximately 11.9% of the age group received regular home care services in 2015 [28]. In the study cohort, the utilization of social and healthcare services, including hospital care, was one of the targets of prospective follow-up. Regular home care was defined as continuous home‑based care provided to clients according to an established care and service plan, with at least one home care visit per week.

Of the 301 participants (59% of the total FIMA cohort) from the Savonlinna region, eight individuals were excluded due to missing baseline data (*n* = 1), absence of follow-up information (*n* = 5), or death shortly after enrollment (*n* = 2).

Preliminary analyses revealed no statistically significant unadjusted associations between randomization status (intervention vs. usual care) and hospitalization outcomes. Therefore, the intervention and control groups were pooled in the present study. In adjusted analyses, the randomization status was still used as a covariate to control its potential associations with explanatory factors within a multivariate analysis.

#### Ethical approval

for the FIMA study protocol was granted by the Research Ethics Committee of the Northern Savo Hospital District and Kuopio University Hospital on February 3, 2015 and the study has been registered with ClinicalTrials.gov (Identifier: NCT02398812, March 2015).

### Data collection

Baseline data collection in 2015 was carried out by interprofessional home care teams consisting of a pharmacist, a physician, and a registered nurse [24, 25]. 

The baseline data collection comprised of participants’ demographic characteristics, documented medical diagnoses, overall comorbidity burden (assessed using the modified Charlson Comorbidity Index, CCI), medication use, and functional assessments. We calculated the CCI using the following diseases with corresponding scores: metastatic or terminal cancer (score of 6), moderate or severe renal insufficiency and non-metastatic cancer (score of 2), coronary artery disease, cerebrovascular disease, dementia of any type, type 1 or 2 diabetes, chronic asthma or obstructive pulmonary disease, peripheral vascular disease, rheumatoid arthritis, or a history of gastrointestinal bleeding (score of 1).

We assessed functional status, physical and cognitive performance, mood, and HRQoL using standardized instruments. Basic and instrumental activities of daily living were evaluated with the Katz Index of Basic Activities of Daily Living (BADL) and the Lawton and Brody Instrumental Activities of Daily Living (IADL) scale; scores range from 0 to 6 and 0 to 8, respectively, with higher scores indicating greater independence. Mobility, lower extremity strength, and balance were assessed using the Timed Up and Go (TUG) test, with longer completion times reflecting poorer mobility. Cognitive function was screened using the Mini-Mental State Examination (MMSE; range 0–30), higher scores indicated better performance. Mood was assessed with the 15-item Geriatric Depression Scale (GDS-15; range 0–15), with higher scores indicating more depressive symptoms.

HRQoL was measured using the EuroQol EQ-5D-3 L index (range − 0.580 to 1.000) and the EuroQol Visual Analogue Scale (VAS; range 0–100), with higher scores indicating better health and quality of life [26, 28].

### Outcome measures

#### Hospitalization

The maximal length of follow-up was one year after the baseline examinations. The study endpoints were dates of death or the end of the follow-up (whichever came first). A total of 25 home care clients died during the follow-up. Participants with nursing home placement (*n* = 27) remained in follow-up for hospitalizations [26]. The follow-up data were collected from the electronic medical records of the East Savo Health Care District. Hospitalizations were recorded as a dichotomic outcome (yes/no) and as a number of hospital days. Inpatient days were presented by setting (primary or secondary care), specialty, and the diagnostic category according to the International Classification of Diseases, Tenth Revision (ICD-10) [29].

Following the collection of clinical and hospitalization data, all personal identifiers were removed to ensure participant anonymity. This de-identification process guaranteed that neither the researchers involved in data analysis and reporting, nor the readers of published findings could identify individual participants.

#### Costs

We calculated the costs of hospitalizations and divided them by person-years. These costs were analyzed across both primary and secondary care settings and by specialties and diagnostic categories. We calculated the costs using the national unit costs of social and health care in Finland compiled by the Finnish Institute for Health and Welfare [30].

Unit costs were adjusted for inflation using the public social and health care expenditure price index for the year 2023 and are presented in euros [31]. For the purposes of this analysis, both tax-funded expenditures and patient fees were aggregated to represent the total cost of hospitalization.

### Statistical analysis

The characteristics of the patients were summarized using percentages, means, medians, standard deviations and quartiles. Mean or median values were compared between groups using a t-test, Mann-Whitney U-test and Pearson χ^2^ -test.

In the main analysis, we applied a two-step procedure known as a hurdle approach developed for modeling positively skewed data with many zeros. First, we used logistic regression to assess factors predicting hospitalizations (yes vs. no) within a year after baseline. Second, we used gamma regression with a log link to assess factors predicting the number of inpatient days. The baseline characteristics presented in Table 1 served as potential factors. The first step included all patients (*n* = 293) whereas the second step included only the hospitalized ones, i.e. those who had been admitted to hospital at least once within the follow-up (*n* = 176).

**Table 1:** Baseline characteristic of the home care clients by number of inpatient days

Variables	Number of inpatient days			All patients
	0 (*n* = 117)	≥ 1 (*n* = 176)	*p*-value	*n* = 293
Age, mean (SD)	81.1 (6.6)	83.6 (5.9)	0.001	82.6 (6.3)
Women, n (%)	79 (67.5)	128 (72.7)	0.361	207 (70.6)
Living alone, n (%)	82 (70.1)	128 (72.7)	0.507	210 (73.4)
Intervention, n (%)	55 (47.0)	92 (52.3)	0.405	147 (50.2)
**Health and diseases**				
Excellent or good self-rated health, n (%)	24 (21.2)	49 (28.7)	0.169	73 (24.9)
Charlson Comorbidity Index, mean (SD)	2.4 (1.8)	3.3 (2.0)	< 0.001	2.9 (1.9)
Diabetes, n (%)	39 (33.3)	66 (37.5)	0.534	105 (35.8)
Dementia, n (%)	22 (18.8)	54 (30.7)	0.029	76 (25.9)
Coronary artery disease, n (%)	48 (41.0)	73 (41.5)	0.518	121 (41.3)
Heart failure, n (%)	27 (23.1)	73 (41.5)	0.001	100 (34.1)
Cerebrovascular disease, n (%)	38 (32.5)	55 (31.3)	0.898	93 (31.7)
Peripheral vascular disease, n (%)	7 (6.0)	24 (13.6)	0.035	31 (10.6)
COPD or asthma, n (%)	16 (13.7)	31 (17.6)	0.419	47 (16.0)
Non-metastatic cancer, n (%)	7 (6.0)	22 (12.9)	0.073	29 (9.9)
Metastatic or terminal cancer, n (%)	< 5	< 5	0.306	4 (1.4)
Previous gastrointestinal bleeding, n (%)	< 5	< 5	0.394	5 (1.7)
Rheumatoid arthritis, n (%)	7 (6.0)	13 (7.6)	0.814	20 (6.8)
Glomerulal filtration rate < 50 ml/min, n (%)	21 (17.9)	58 (33.0)	0.005	79 (27.0)
**Medication**				
Number of all medicines, median (IQR)	14 (11.0, 17.0)	15 (12.0, 18.0)	0.297	14.0 (12.0, 18.0)
≥ 10 medicines in use, n (%)	103 (88.0)	153 (86.9)	0.859	256 (87.4)
Class D medicine in the Meds75+, n (%)	35 (29.9)	38 (21.6)	0.129	73 (24.9)
**Functioning and mood**				
MMSE, median (IQR)	23 (20.0, 26.0)	23 (20.0, 25.0)	0.079	23.0 (20.0, 26.0)
BADL, median (IQR)	5 (5.0, 6.0)	5 (4.0, 6.0)	< 0.001	5.0 (4.0, 6.0)
IADL, median (IQR)	5 (3.0, 6.0)	4 (2.0, 6.0)	< 0.001	4.0 (2.0, 6.0)
TUG, second, median (IQR)	18.6 (12.8, 26.9)	22.3 (16.0, 33.9)	0.015	20.4 (14.7, 29.9)
GDS-15, median (IQR)	4 (2.25,7.0)	5 (3.0, 7.5)	0.258	4.0 (3.0, 7.0)
**Health related quality of life**				
Equation 5D, median (IQR)	0.69 (0.59, 0.73)	0.59 (0.52, 0.73)	0.006	0.6 (0.5, 0.7)
Quality of life VAS 0-100, median (IQR)	60.0 (50.0, 73.0)	55.00 (48.5, 69.5)	0.008	55.0 (49.0, 70.0)

Received medication assessment intervention, COPD Chronic obstructive Pulmonary Disease, Meds75 + database of medication for older adults, class D: avoid use in older adults, MMSE Mini-Mental State of Examination, BADL Basic Activities of Daily Living, IADL Instrumental Activities of Daily Living, TUG Timed Up and Go test, GDS-15 Geriatric Depression Scale, EQ-5D EuroQol instrument with 5 dimensions and 3 levels for measuring health-related quality of life, P-value threshold for statistical significance: < 0.05.

The number of inpatient days was expressed relative to the length of follow-up, i.e., transformed to a number per year for each patient regardless of the actual length of the follow-up, which for some patients was shorter than one year. In both steps, we first analyzed every factor separately, adjusting for age, sex, and the intervention group (medication assessment or usual care). Secondly, we constructed multivariate logistic and gamma regressions based on factors related to health and functioning that showed statistical significance (*p* < 0.05) in the adjusted univariate analyses. Instead of single diseases, we used CCI as an explanatory factor in multivariate regressions, since the number of cases per disease varied a lot from disease to disease, and for some diseases the number was very low (< 5). Furthermore, CCI combines the information on diseases recorded at baseline evaluations. The number of medicines reflects also multimorbidity and disease burden and was therefore excluded from multivariate regressions. Regarding logistic regressions, Area Under the Curve (AUC) was calculated to estimate performance, and the Hosmer and Lemeshow test was executed to evaluate the goodness-of-fit.

The significance of the results is presented as p-values, and values < 0.05 are considered statistically significant. All statistical analyses were performed using IBM SPSS software version 29).

**Fig. 1:**
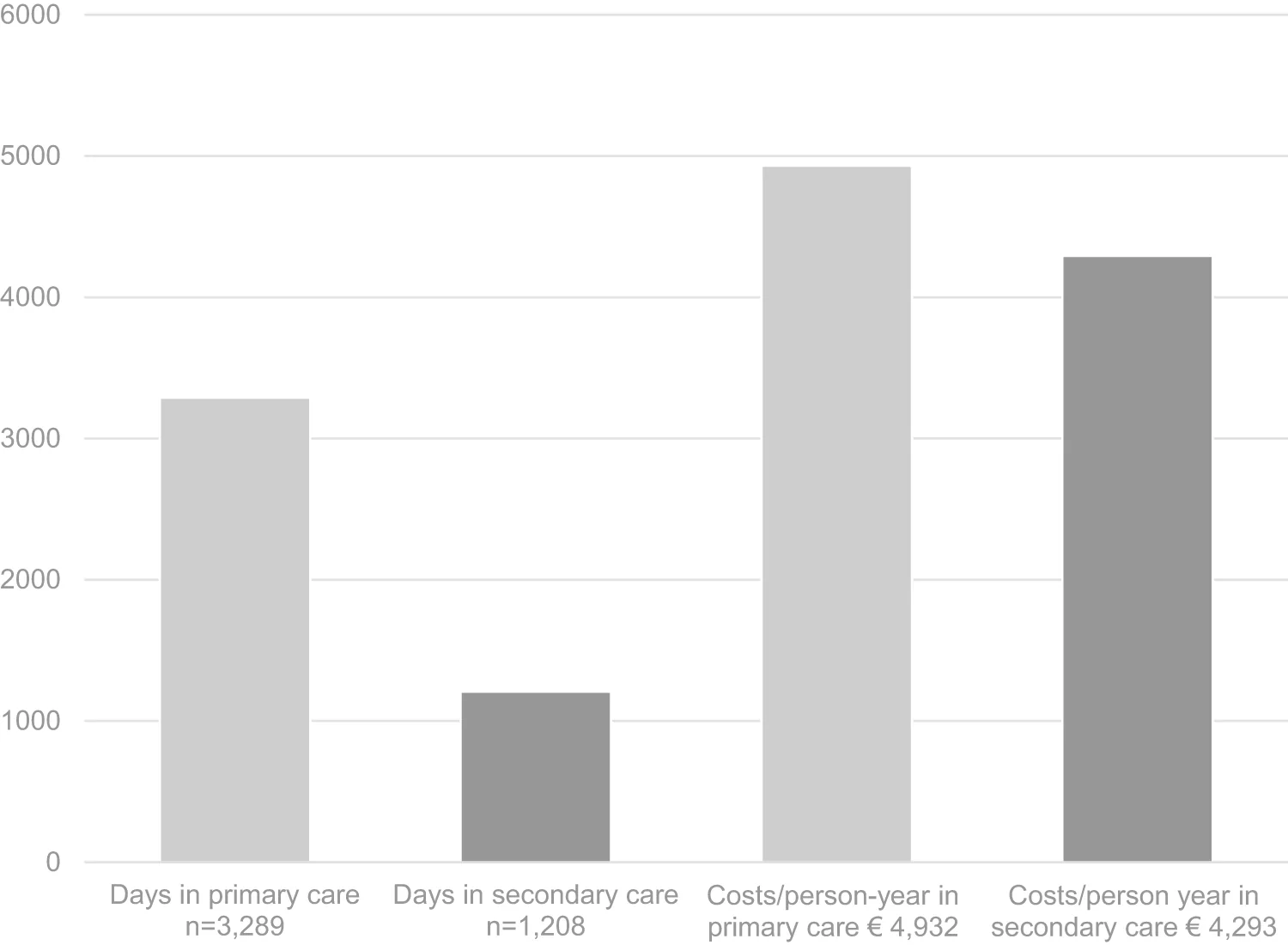
Days and costs of hospitalizations in primary and secondary care among 293 home care clients

## Results

At the baseline (Table 1), the mean age of all the clients (*n* = 293) was 82.6 years (range 65–95 years). The majority were women and lived alone. The most common chronic conditions were coronary artery disease (41%), diabetes (36%), heart failure (34%), cerebrovascular disease (32%), renal failure (27%) and dementia (26%).

The mean CCI of all clients was 2.9. Most clients had difficulties in performing daily activities; half of the home care clients needed help with at least one basic and four instrumental activities of daily living. The MMSE scores were indicative of mild or more advanced dementia in half of the home care clients (median MMSE score 23.0). The median time to complete the TUG test was 20.4 s.

A total of 176 (60%) home care clients were hospitalized at least once during the follow-up. The home care clients with hospitalizations were older, had a higher CCI and suffered more often from dementia, peripheral vascular disease or heart or renal failure at the baseline of the study compared with home care clients without hospitalizations. They also had more difficulties in BADLs and IADLs, worse TUG times, and lower HRQoL than the home care clients without hospitalizations.

### Factors predicting hospitalization

Logistic regression showed that prior heart failure was associated with almost two-fold odds of hospitalization and peripheral vascular disease with three-fold odds (Table 2). The other factors statistically significantly associated with hospitalizations were CCI, renal failure, BADL and IADL scores, TUG times, and HRQoL.

**Table 2:** Factors associated with hospitalization. Logistic regression in which each variable was analyzed separately. All the analyses were adjusted for age, sex and intervention group

Variables	B	*p*-value	OR (CI95%)
Living alone, n (%)	-0.24	0.396	0.79 (0.46–1.37)
**Comorbidity burden**			
Charlson Comorbidity Index (0–19)	0.23	0.001	1.26 (1.10–1.45)
**Health and diseases (yes/no)**			
Excellent or good self-reported health	-0.39	0.184	0.68 (0.38–1.21)
Diabetes	0.29	0.256	1.34 (0.81–2.23)
Dementia	0.55	0.063	1.73 (0.97–3.07)
Coronary artery disease	-0.05	0.849	0.95 (0.59–1.55)
Heart failure	0.72	0.009	2.06 (1.20–3.53)
Cerebrovascular disease	0.04	0.897	1.04 (0.61–1.75)
Peripheral vascular disease	1.01	0.027	2.74 (1.12–6.73)
COPD or asthma	0.38	0.266	1.47 (0.75–2.87)
Non-metastatic cancer	-1.49	0.207	0.23 (0.02–2.27)
Metastatic or terminal cancer	0.70	0.124	2.02 (0.82–4.96)
Previous gastrointestinal bleeding	-0.53	0.572	0.59 (0.09–3.71)
Rheumatoid arthritis	0.17	0.736	1.18 (0.44–3.16)
Glomerulal filtration rate < 50 ml/min	-0.62	0.041	0.54 (0.30–0.98)
**Medication**			
Number of all medicines	0.02	0.311	1.03 (0.98–1.08)
Class D medicine in the Meds75+	-0.51	0.068	0.60 (0.82–2.16)
≥ 10 medicines in use	-0.08	0.825	0.92 (0.45–1.90)
**Functioning and mood**			
MMSE (0–30)	-0.03	0.267	0.97 (0.92–1.02)
BADL (0–6)	-0.33	0.003	0.72 (0.59–0.89)
IADL (0–8)	-0.21	< 0.001	0.81 (0.72–0.92)
TUG, second	0.02	0.047	1.02 (1.00–1.03)
GDS-15 (0–15)	0.04	0.280	1.04 (0.97–1.13)
**Health related quality of life**			
Equation 5D (-0.580-1.000)	-1.39	0.009	0.25 (0.09–0.71)
Quality of life VAS 0-100	-0.02	0.014	0.98 (0.97–1.00)

B coefficient per one unit increase, COPD Chronic obstructive Pulmonary Disease, Meds75 + database of medication for older adults, class D: avoid use in older adults, MMSE Mini-Mental State of Examination, BADL Basic Activities of Daily Living, IADL Instrumental Activities of Daily Living, TUG Timed Up and Go test, GDS-15 Geriatric Depression Scale, EQ-5D EuroQol instrument with 5 dimensions and 3 levels for measuring health-related quality of life, P-value threshold for statistical significance: < 0.05.

In the multivariate logistic regression, higher CCI (*p* = 0.008) predicted hospitalization whereas age (*p* = 0.101), sex (*p* = 0.329), intervention (*p* = 0.253), BADL (*p* = 0.571), IADL (*p* = 0.225), TUG (*p* = 0.293), EQ-5D-3 L (*p* = 0.372) did not. The model correctly classified 66% of clients who were hospitalized but only 46% of those who were not (AUC = 68.2). The Hosmer and Lemeshow test indicated a good fit of the model (*p* = 0.700).

### Factors predicting inpatient days

Among the hospitalized home care clients (*n* = 176), history of diabetes, dementia, heart failure or terminal cancer, number of medicines and MMSE, BADL and IADL scores were associated with the cumulative number of inpatient days in age, sex and intervention status adjusted univariate analyses (Table 3). The presence of dementia was associated with an almost two-fold increase in the number of hospital days. Each one-point increase in BADL, IADL, and MMSE scores were associated with a lower number of inpatient days. Diagnosed diabetes, heart failure, terminal cancer, and a higher number of medicines were associated with a decreased number of hospital days.

**Table 3:** Baseline characteristics associated with the cumulative number of inpatient days per month. Gamma regression with a log link in which each variable was analyzed separately. All the analyses were adjusted for age, sex and intervention group

Variables	e^B^ (CI 95%)	*p*-value	
Living alone, n (%)	1.34 (0.88–2.04)	0.174	
**Comorbidity burden**			
Charlson Comorbidity Index	0.98 (0.90–1.06)		0.553
**Health and diseases**			
Excellent or good self-reported health	0.84 (0.56–1.25)	0.395	
Diabetes	0.62 (0.42–0.89)	0.010	
Dementia	1.90 (1.29–2.82)	0.001	
Coronary artery disease	0.77 (0.53–1.10)	0.149	
Heart failure	0.64 (0.44–0.92)	0.016	
Cerebrovascular disease	0.86 (0.59–1.27)	0.456	
Peripheral vascular disease	0.90 (0.53–1.52)	0.692	
COPD or asthma	0.64 (0.40–1.03)	0.067	
Non-metastatic cancer	1.00 (0.58–1.74)	0.997	
Metastatic or terminal cancer	0.02 (0.00-0.19)	< 0.001	
Previous gastrointestinal bleeding	0.35 (0.06–1.94)	0.229	
Rheumatoid arthritis	1.26 (0.63–2.50)	0.518	
Glomerular filtration rate < 50 ml/min	1.04 (0.65–1.41)	0.831	
**Medication**			
Number of all medicines	0.96 (0.93–0.99)	0.015	
Class D medicine in the Meds75+	1.02 (0.66–1.59)	0.928	
≥ 10 medicines in use	0.78 (0.46–1.33)	0.357	
**Functioning and mood**			
MMSE	0.93 (0.90–0.97)	0.001	
BADL	0.86 (0.76–0.97)	0.017	
IADL	0.86 (0.79–0.93)	< 0.001	
TUG	1.01 (1.00-1.01)	0.293	
GDS-15	1.03 (0.97–1.10)	0.299	
**Health related quality of life**			
EQ-5D-3 L	0.91 (0.47–1.79)	0.793	
Quality of life VAS 0-100	0.99 (0.98-1.00)	0.158	

COPD Chronic obstructive Pulmonary Disease, Meds75 + database of medication for older adults, class D: avoid use in older adults, MMSE Mini-Mental State of Examination, BADL Basic Activities of Daily Living, IADL Instrumental Activities of Daily Living, TUG Timed Up and Go test, GDS-15 Geriatric Depression Scale, EQ-5D EuroQol instrument with 5 dimensions and 3 levels for measuring health-related quality of life, P-value threshold for statistical significance: < 0,05.

In the multivariate gamma regression with a log link, MMSE (e^B^ 0.95, CI95% 0.91-1.00, p-value 0.039) predicted the number of hospital days. Higher MMSE scores were associated with fewer days, whereas intervention status, age, sex, CCI, BADL and IADL were not significant explanators (*p* > 0.05) of hospital days.

### Reasons for and costs of hospitalizations

During the follow-up period, the home care clients were hospitalized for 4,697 days; 3,489 in primary care and 1,208 in secondary care hospitals (Table 4). There were 19.3 hospital days per person-year in the entire cohort, 13.6 in primary care and 5.7 in secondary care. The study participants did not have any tertiary care hospital admissions during the follow-up.

**Table 4:** Days and costs of hospitalizations

		*n* (%)	Days/person-year	Total costs (€)	Cost/person-year (€)
**Total**		**4,697 (100)**	**19.28**	**2,370,910**	**9,225**
**Primary care**		**3**,**489 (74.3)**	**13.61**	**1**,**267**,**519**	**4**,**932**
**Secondary care**		**1**,**208 (25.7)**	**5.71**	**1**,**103**,**391**	**4**,**293**
Main specialties:	Internal medicine	503 (41.6)	1.96	350,669	1,368
	Surgery	500 (41.4)	1.95	679,435	2,616
	Pulmonary disease	120 (9.93)	0.47	66,271	258
	Neurology	52 (4.30)	0.20	24,919	97

In secondary care, most patients were treated in internal medicine (42%) or surgery wards (41%). The most common diagnostic categories for all inpatient days were diseases of the circulatory system (ICD-10 I00-I99, 25% of the days), injuries, poisonings and other consequences of external causes (S00-T98,16%), as well as diseases of the nervous system (G00-G99,15%).

The total cost of hospitalizations was €2,370,910, with primary care accounting for €1,267,519 and secondary care for €1,103,391 of the cost. In the entire cohort, the costs were €9,225 per person-year, €4,932 from primary care and €4,293 from secondary care hospitalizations (Figure 1). The costs of primary and secondary care hospitalizations were nearly similar, even though the number of inpatient days in primary care was three times higher than in secondary care. Internal medicine and surgery costs were €1,368 and €2,616 per person-year, respectively.

## Discussion

### Key findings

In this prospective study, hospitalizations among home care clients were associated with a greater comorbidity burden, a history of peripheral vascular disease or heart or renal failure, and worse mobility. Better functioning in basic and instrumental activities of daily living and a higher quality of life decreased the odds of hospitalization. The primary reasons for hospitalizations most often fell into the diagnostic categories of circulatory system diseases, injuries and nervous system diseases. The home care clients were hospitalized on average for 19 days per year and hospitalization costs per client exceeded €9,000 per year. The number of primary care inpatient days was nearly three times higher than that of secondary care, yet the hospitalization costs were nearly the same. Rehabilitation of older patients is mainly conducted in primary care hospitals, which may partly explain prolonged hospital stays. Moreover, the recovery process is often extended if baseline functional capacity is reduced. On the other hand, all the patients cannot be discharged back to their own homes, and they usually have to wait for a nursing home place in primary care wards.

### Comparison with previous studies

In our study, 60% of home care clients were hospitalized within a one-year time frame. This is more than in an earlier Finnish study, where 43% of home care clients were hospitalized during a one-year follow-up [4, 5]. This may be due to study designs, the reference study included new home care clients only and in addition our inclusion criteria selected to identify home care clients with medication-related problems.

In this home care cohort, a higher comorbidity burden and a history of heart or renal failure and peripheral vascular disease increased the odds of hospitalization. A large register-based study conducted by the inter-RAI network reported that coronary artery disease, heart failure, cancer, emphysema and renal failure increased the six-month hospitalization risk among older home care clients in Canada, Finland and the U.S [1]. Furthermore, in a large register-based U.S. cohort of new Medicare Home Health (MHH) clients aged ≥ 65 years, a primary diagnosis of heart disease was associated with a higher risk of hospitalization within two months [18]. In an earlier MHH cohort, congestive heart failure increased the hospitalization risk by 42% when it was identified as the primary diagnosis for receiving home care services [17]. Similar to our findings, an Israeli study of 1,932 home care clients aged ≥ 70 years reported that a higher comorbidity burden was a risk factor for hospitalizations within the subsequent year [32]. An Italian study of older home care clients (*n* = 1,291) also reported that comorbidity raised the one-year hospitalization risk [11].

In this study, higher HRQoL decreased the odds of hospitalization. In contrast, there were no differences in self-rated health between those who were hospitalized and those who were not. Previously, HRQoL and self-rated health have been associated with emergency department (ED) visits and hospitalizations among home care clients [5, 33]. A Finnish study showed that poor self-rated health was an independent risk factor for unplanned hospitalizations among new home care clients [5]. An Australian study of 1,999 chronically ill patients (mean age 63 years) reported that after one and two years, lower HRQoL was associated with an approximately two-fold increase in the number of ED visits and hospitalizations [21]. Lower HRQoL also increased the risk of unplanned hospital readmissions in chronically ill home-dwelling older Australians [20].

Earlier U.S. studies found that depressive symptoms were associated with hospitalizations among older home care clients [17, 18] whereas in our study, GDS-15 scores showed no association with hospital use. In the U.S. studies, depressive symptoms were assessed using a two-item questionnaire: whether or not the client had a depressed mood (feeling sad or tearful) or by using the two-item Patient Health Questionnaire. The difference between study results may be due to different measurement methods.

In the present study, longer TUG times and lower BADL and IADL scores had correlative associations with hospitalizations. In addition, lower BADL, IADL and MMSE scores were also associated with a higher number of inpatient days. An earlier Finnish study found that cognitive impairment increased the risk of hospitalization among new home care clients aged ≥ 65 years [4]. Impaired mobility itself and as a manifestation of frailty syndrome may increase vulnerability during acute illnesses and delay recovery [34]. Injuries were the second leading cause of hospitalizations in our study, and most injuries of older people are fall-related [35]. A Swedish study of 1,402 older people showed that ADL dependency increased hospital admissions in six-year follow-up [15]. Functional dependency also increased the use and costs of other health care services, as reported in a U.S. study of community-dwelling older adults [22]. The excess health care costs were approximately $10,000 per dependent person over a period of two years compared to those who remained independent.

In our study, diabetes, heart failure, terminal cancer and greater number of medicines were associated with fewer inpatient days. This finding differs from earlier studies. It can be assumed that these clients had a higher baseline frequency of home care visits, which could have facilitated a more rapid discharge from the hospital.

In this study, based on multivariate logistic regression, the CCI had direct association with hospitalizations and in multivariate gamma regression MMSE was associated directly with inpatient days. In our dataset, functional impairment may exert its effect on the outcomes indirectly through the CCI, and the MMSE scores may provide a more accurate representation of cognitive performance than dementia diagnoses.

Overall, baseline characteristics explained only a small share of inpatient days in the present home care cohort. There might be organizational factors, for example a lack of care services required after discharge, that affect the duration of hospitalizations more than the patient-related factors. It would be important to identify these factors as well.

The annual costs of hospitalizations were on average €9,250 per home care client. The costs were not evenly distributed in the cohort, as 42% of the home care clients were not hospitalized during the follow-up. In addition, the primary care and secondary care costs were almost the same, even though only a quarter of the inpatient days were from secondary care. The average cost of one inpatient day was €363 and €913 in primary and secondary care hospitals, respectively. Treatment costs in English geriatric wards and Finnish primary care wards were comparable in 2010–2011 (€238 vs. €213 per patient per day) [19].

The most common reasons for hospitalizations were treatment of circulatory system diseases, injuries and nervous system diseases. A recent Finnish study reported that infectious diseases were the most common reason for hospitalizations among home care clients [4]. In the present analysis, infections were classified under several ICD-10 categories (for example pleuropulmonary infections under category J and urinary tract infections under N). This might explain the difference.

A meta-analysis of 20 studies assessing the impact of home care on the use of inpatient care reported that home care reduced hospital days [3]. Home care nurses can identify symptoms of acute illnesses and exacerbations of chronic conditions in their early stages; in addition, some of these conditions can be managed at home as well. However, this requires the availability of skilled nursing staff and sufficient financing, both of which seem to be challenges for aging societies, including Finland. The proportion and more recently also the absolute number of older Finns receiving regular home care services have decreased [35].

### Strengths and limitations

One of the strengths of the present study was that the study was carried out in the real-life context. Data collection was conducted using validated instruments appropriate for older people. The home care clients were interviewed and examined instead of leaning solely on the register-based data. The records of hospitalizations can be assumed to be highly accurate, because the data collection is obligatory and regulated by law and it serves also as a basis for invoicing.

Nonetheless, certain limitations must be acknowledged. Predicting hospitalization and the duration of inpatient care among home care clients remains challenging. This unpredictability may stem not only from sudden and acute changes in health status, but also from structural and functional complexities of the health and social care services. The relatively small sample size may have reduced the statistical power of the present study. On the other hand, statistically significant findings observed in a small sample are likely to reflect true effects Furthermore, the use of six or more medicines daily was a eligibility criteria of FIMA study and might have enriched people with polypharmacy to the study participants. The dataset was derived from a single geographic region in Finland. The participants represented approximately 20% of all regular home care clients in the study area [24, 25]. Nevertheless, due to the implementation of national quality guidelines for home care, we consider the findings to be generalizable to the wider Finnish home care context and are potentially applicable to other Nordic countries with comparable service structures.

## Conclusions

A higher comorbidity burden, a history of peripheral vascular disease or heart or renal failure, functional impairment and worse mobility were predictors of hospitalizations. Better functioning in basic and instrumental activities of daily living and higher health-related quality of life decreased the odds of hospitalization among home care clients. Functional and cognitive impairments were associated with more inpatient days. In addition to optimal disease management, preserving functional capacity of home care clients is a critical component of comprehensive care.

Annual hospitalization costs per home care client exceeded €9,000. The costs of primary and secondary care hospitalizations were nearly similar, even though the number of inpatient days in primary care was three times higher than that in secondary care. The most common primary reasons for hospitalizations were circulatory system diseases and injuries, which could serve as primary targets in the prevention of hospitalizations.

Early detection of emerging risks, appropriate management of chronic conditions and preventive interventions suitable for frail older adults should be promoted in home care. For example, enhanced home-based monitoring of cardiovascular conditions, effective management of cognitive disorders, and improved prevention of accidents, most notably falls occurring at home, may reduce hospital admissions and shorten inpatient stays.

## Data Availability

The datasets generated and/or analyzed during the current study are not publicly available due to participants not consenting to sharing datasets publicly; however, they are available from the corresponding author on reasonable request.
